# Interactive Quiz-Based Anatomy Teaching for Medical Undergraduate Students

**DOI:** 10.7759/cureus.52353

**Published:** 2024-01-16

**Authors:** Akanksha Verma, Navbir Pasricha, Amit Chaudhary, Rajan Bhatnagar, Eti Sthapak, Anamika Gaharwar

**Affiliations:** 1 Anatomy, Dr. Ram Manohar Lohia Institute of Medical Sciences, Lucknow, IND; 2 General Medicine, T.S. Misra Medical College and Hospital, Lucknow, IND

**Keywords:** medical device technology development and assessment, medical education, learning outcome, interactive quiz based lecture, didactic lecture

## Abstract

Background: Anatomy is one of the most volatile subjects and needs the learner to understand and retain a lot of information and terms. It is thus very important to continuously upgrade the methodology from the traditional didactive to interactive teaching to make the student an active learner and engage him in the learning process to categorize and analyze anatomical facts and knowledge.

Aims and objectives: The study was done to compare the learning outcomes and perception of medical students towards didactic lectures and interactive quiz-based lectures in anatomy.

Methodology: The study was conducted amongst the 200 Year 1 medical undergraduate students enrolled in the Department of Anatomy at Dr. Ram Manohar Lohia Institute of Medical Sciences, located in Lucknow, India. The 200 students comprised 120 males (60%) and 80 females (40%). The mean age of male students was 19.67 years and of females was 19.52 years. The students were divided into two groups of hundred students each by a method of convenience sampling. Students of group I were taught by an interactive quiz-based lecture and group II by a traditional didactic lecture. A pre- and post-test were conducted for both groups and feedback for both methods was taken by using a pre-validated feedback form based on a 5-point Likert scale.

Results: On statistical analysis, it was found that in the post-test the performance of group I taught by the interactive quiz-based study was better as compared to group II taught by traditional didactive teaching, but was not statistically significant (p=0.233, p>0.05). The feedback from students revealed that 45.9% of them strongly agreed and 44.9% agreed with the fact that quiz-based lectures are better than routine lectures.

Conclusions: Results of the present study clearly indicate that the introduction of quiz-based anatomy teaching for undergraduate medical students was well received and appeared to improve their learning outcomes in the form of increased attention and participation during class and would lead to better retention of the topics taught during interactive lectures. To the best of our knowledge, no previous study has been done to document the efficacy of quiz-based teaching for the subject of anatomy.

## Introduction

In the past few years, the widespread adoption of Curriculum-Based Medical Education (CBME) and the advent of newer techniques and research on many innovative methods of teaching and learning have started modifying medical education worldwide [1]. For teaching large groups of students, most of the professional colleges in India adopt traditional or didactic lectures [2]. In the present scenario, these lectures are not suitable for teaching each and every student and situation. Usually, didactic lectures are of 1 hour to 40 minutes duration, in which it is difficult to continually keep the student engaged as the attention span wanes around after 20 minutes [2]. In routine didactic lectures, students are not actively involved in the process of learning, and they only act as passive receivers of information [3]. It is a well-known fact that learning is an active process and interactive teaching-learning methods are considered best educational practice [4].

With interactive lectures, there is some kind of interaction among teachers and students during the lecture [1]. There can be many kinds of interaction during the class out of which some commonly used ones are questioning the students, quizzes, using audience responses, role play, case studies, playing relevant audiovisual materials, etc. Interactivity during the lecture breaks the monotony of the class and increases the level of student’s attention [2]. Interactive teaching-learning methods are highly important for developing critical thinking among medical students and also for their future clinical practices as they learn to analyze concepts gained during the lecture [5,6]. Anatomy is one of the important subjects of basic medical science, but it is highly volatile and needs the learner to understand and retain a lot of information and terms, in a very limited time duration. It is highly recommended to change the methodology from conventional to interactive teaching and learning to make the subject interesting and to intricate the habit of active learning among students. The present study was conducted to assess and compare the learning outcomes and perception of medical students towards didactic lectures and interactive quiz-based lectures in anatomy. It was conducted with the objectives of evaluating the gain in knowledge with interactive quiz-based lectures, comparing the effect of these methods on the performance of students, and obtaining the student's feedback regarding interactive quiz-based lectures.

## Materials and methods

A case-control analytical study was conducted on 200 first-year medical undergraduate students of Dr. Ram Manohar Lohia Institute of Medical Sciences, Lucknow, Uttar Pradesh. The 200 students comprised 120 males (60%) and 80 females (40%), The mean age of male students was 19.67 years and of females was 19.52 years. The study was conducted in the Department of Anatomy after obtaining ethical approval from the institutional ethics committee (IEC No. 4/21). Because the study was done during covid pandemic all the sessions were conducted online on the Zoom platform. The entire batch of 200 students was divided into two groups according to their class roll numbers. Group I was the study group (lecture with quiz) which had 100 students from Roll Numbers 1 to 100 and group II comprised the Control group taught by traditional didactic lecture which also had 100 students from Roll Numbers 101 to 200. The topic for the lecture selected was the gross anatomy of the heart. This topic was selected as it is an important topic for medical undergraduates and is required for a basic understanding of all medical specialties. At first, a pre-test was conducted for both groups, consisting of pre-validated multiple-choice questions, henceforth the students of group I were taught by a lecture with a quiz, and group II was taught by a didactic lecture. The quiz was conducted after every 15 minutes in a 60-minute lecture. The quiz was conducted by the first author by asking one-word cognition-based and anatomical basis analytical-type questions. A post-test was conducted after the completion of the topic for both groups. Perception of students towards quiz-based lectures was also assessed by using a prevalidated feedback form based on a 5-point Likert scale. All the pre-test, post-test, and feedback forms were prepared on Google Forms.

The efficacy of conducting a lecture with a quiz and didactic lecture was compared by using pre and post-test scores and the perception of students toward the new method of the lecture was assessed by responses provided in the anonymized structured feedback form. Statistical analysis was done by using Statistical Package for Social Sciences (SPSS) Version 20 (IBM Corp., Armonk, NY), Statistical Analysis Software.

## Results

Efficacy of lecture with quiz and didactic lecture

Among the total number of 200 students of MBBS Phase I, only 129 students (58 in the study group and 71 in the control group) were able to submit their both pre-test and post-tests during their online sessions (Table 1).

**Table 1:** Total number of students included in the study

Group	Number of students (N)	Percentage
Group I [Lecture with quiz]	58	45.0
Group II [Didactic lecture]	71	55.0
Total	129	100.0

Out of 200 students included in the study, only 129 gave both pre-test and post-test and their marks were thus included in the data. The mean scores of pre- and post-tests of group I (lecture with quiz) were 5.7069 and 8.137 and of group II (didactic lecture) were 5.2394 and 7.6901. On application of paired t-test for significance, differences in the mean scores of pre-tests and post-tests of both the groups were compared and found to be significant (p-value < 0.001) (Table 2).

**Table 2:** Comparison between pre-test and post-test score of both groups Applied paired t-test, p-value < 0.05 is significant

Groups		Mean score	N	Std. Deviation	P-value
Group I (lecture with quiz	Pre test	5.7069	58	1.68594	<0.001
	Post test	8.1379	58	1.71114	
Group II (didactic lecture)	Pre test	5.2394	71	2.07340	<0.001
	Post test	7.6901	71	2.01134	

By applying an unpaired t-test, we have compared the post-test scores of group I and group II, and the difference was not significant (p-value = 0.233) but the mean score of group I (lecture with quiz) students was higher than group II (didactic lecture) students (Table 3).

**Table 3:** Comparing post test scores of group I and group II Applied unpaired t test, p-value < 0.05 is significant

Group	N	Post test Mean score	Std. Deviation	P-value
Group I (Lecture with quiz)	58	8.1379	1.71114	0.233
Group II (Didactic lecture)	71	7.6901	2.01134	

Perception of students toward quiz-based lectures was assessed by using a pre-validated feedback form based on a 5-point Likert scale having nine close-ended and three open-ended questions. Table 4 shows the responses of a total of 98 students (out of 200 students attending both types of teaching) who gave their feedback regarding the new intervention after both the sessions and for all the nine variables the students supported interactive lectures more than the routine purely didactic lectures.

**Table 4:** Feedback responses of students SA - Strongly agree, A - Agree, N - Neutral, D - Disagree, SD - Strongly disagree All values are in percentage

SN	Close-ended questions	SA	A	N	D	SD
1.	Quiz based lecture is better than routine lecture	45.9	44.9	9.2	0	0
2.	Your knowledge increased post session	41.8	53.1	5.1	0	0
3.	Session made the topic more interesting	37.8	48.0	13.3	0.9	0
4.	Session made you attentive and motivated to learn the topic	37.8	46.9	14.3	1	0
5.	Recall of the topic is better by this session	36.7	57.1	6.2	0	0
6.	Session was interactive	32.7	51.0	13.3	3.0	0
7.	Session has developed motivation in learning anatomy	35.7	46.9	14.3	3.1	0
8.	This type of session can be used for other subjects of basic sciences	36.7	48.0	13.3	0	2.0
9.	Session will be helpful in learning of clinical sciences in future	39.8	43.9	13.3	2.0	1.0

## Discussion

The present study employed a quiz during a lecture as an interactive teaching-learning tool compared its efficacy with a didactic lecture and documented that the average marks obtained by students having a lecture with a quiz (group I) were better than those of group II (students having a didactic lecture), and although the difference was not found to be significant the student’s feedback clearly showed that they found quiz based lecture more stimulating than routine didactic lectures with respect to various parameters as it made the topic interesting, recall of the topic was better, they felt more attentive and motivated for self-learning, etc. Results of the study indicate that the introduction of quiz-based anatomy teaching was well received and appeared to improve their learning outcomes in the form of increased attention and participation during class. To the best of our knowledge, no previous study has been done to document the efficacy of quiz-based teaching for the subject of anatomy.

It is well known that the didactic lectures are the most commonly used but also the highly criticized, traditional way of teaching large groups worldwide. They can lead to a syndrome known as “lecturalgia” (painful lecture) that is characterized either by a state of heightened emotions (e.g., agitation, frustration, and anger) or suppressed emotions (e.g., apathy and somnolence). This condition of lecturalgiacan occurs commonly because of three things - the audience, the setting, and the lecturer. However, it is seen that usually the fault lies on the side of lecturers [7]. To prevent this and to plan effective lectures, the Association for Medical Education in Europe guide for lecturers provides a good outline of the art of lecturing [8].

Traditional lectures are good for delivering a large amount of information in a short time, but they can be boring and cause people to lose concentration because of a lack of active participation [2]. Interactive teaching can capture the student’s attention and make them more receptive and involved with the contents of the lecture [9]. Active learning promotes better understanding and retention of topics, development of communication skills, and better clarification of doubts, and ultimately leads to better reproducibility in the future [10]. Most of the disadvantages associated with didactic lectures are addressed by using different interactive teaching-learning methods and it is associated with increased satisfaction for both teacher and learner [11].

During the designing of the interactive lecture, we have to make sure about two things primarily [12] that the lecture should be divided into segments, and some activities should be created for students’ active participation. There are many kinds of activities that can be used during lectures to increase students' involvement like creative tasks, games (role plays, imitations), use of human resources (inviting experts), use of new materials (audio-visual materials, asking questions, quiz), solving tasks (brainstorming, case analysis), etc. [13].

There are various studies done in the past that positively support our study. A feedback questionnaire study conducted by Kaur et al., with undergraduate medical students after using various interactive techniques for teaching, reflected that student preferred to be taught by different interactive teaching techniques rather than didactic lectures [14]. Chilwant, in his study with 75 second-year medical students, compared the structured interactive lectures with conventional lectures in pharmacology by giving questionnaires and multiple-choice question tests and found that only 3% of students were willing to continue with conventional teaching and the majority were in favor of interactive methods [1]. Roopa et al., in 2013, conducted a study on 78 first-year undergraduate dental students in physiology. They conducted 12 alternate interactive and regular lectures on the cardiovascular system and at the end obtained students' feedback with the help of a structured 5-point Likert scale questionnaire. Approximately 92% of students were in favor of interactive lectures and only 21% preferred regular lectures for keeping them attentive [2]. A study was conducted by Gupta et al., in 2015, with 150 students of second professional medical students on the subject of pharmacology. Students were divided into two groups each of 75 students. Total eight structured interactive sessions in the form of a quiz, case-based scenario, think pair and share, and role play were conducted with both groups, and it was seen that more than 75% of students favored interactive teaching and quiz was the most preferred method of interaction out of all chosen methods for interaction [10]. An interventional study done by Begun et al., for undergraduate students in community medicine, used four different interactive teaching-learning methods (think-pair-share, buzz session, case-based learning, and pass the problem found that approximately 92% of students were satisfied with the introduction of interactive teaching-learning in community medicine and pass the problem was the most (67.86% students) liked method [15]. Verma et al. suggested the use of technology-like cues incorporated in PowerPoint presentations and non-technology-based interactive techniques to enhance the learning experience [16].

We can safely infer that incorporating interactivity in the didactic lecture makes the content more learner-centric and increases learner concentration and participation, but it requires more input from the facilitator [1]. The facilitator needs more time and resources for prior planning with the interactive method being used apart from overcoming the fear of losing control over the class or the fear of not being able to deliver the topic completely [16]. With interactive teaching methods like quiz-based teaching, the facilitator can ignite the minds of the learners, allowing them to think and analyze a problem and further continue with peer-based and self-directed learning even if the topic is not completed in the class session.

Limitations

All the sessions were conducted online so there were technical issues in joining and response submission for the students. Also, there is the inclusion of only one interactive teaching-learning method in the form of quiz-based lectures. Despite its limitations, the study exhibits strength in the fact that students strongly appreciated the quiz-based interactivity during the lecture, which inspired the authors to take it up as a part of routine didactic teaching.

## Conclusions

The results of the present study clearly showed that students preferred interactive quiz-based lectures more than didactic lectures in Anatomy. Interactivity creates a lively and enjoyable atmosphere during the lectures motivates students to actively participate and learn during class and enhances communication skills among students It is very important for medical students for their future clinical practices. It can be perceived that this would lead to better outcomes in the form of long-term retention and analysis of the topic. To the best of our knowledge this is the first study that attempts to analyze the efficacy of using quiz-based interactivity in anatomy lectures and on the basis of the feedback received the authors recommended that medical teachers should start including some form of interactivity in didactic lectures to enhance learning and retention among the students.

## References

[1] Chilwant KS (2012). Comparison of two teaching methods, structured interactive lectures and conventional lectures. Biomed Res.

[2] Roopa S, Geetha M B, Rani A, Chacko T (2013). What type of lectures students want? - a reaction evaluation of dental students. J Clin Diagn Res.

[3] Byrne PS, Harris CM, Long BE (1976). Teaching the teachers. Med Educ.

[4] Butler JA (1992). Use of teaching methods within the lecture format. Med Teach.

[5] Rao SP, DiCarlo SE (2000). Peer instruction improves performance on quizzes. Adv Physiol Educ.

[6] Saroyan A, Snell L (1997). Variations in lecturing styles. Higher Educ.

[7] McLaughlin K, Mandin H (2001). A schematic approach to diagnosing and resolving lecturalgia. Med Educ.

[8] Brown G, Manogue M (2001). Amee Medical Education Guide No. 22: refreshing lecturing: A guide for lecturers. Med Teach.

[9] Pettit RK, McCoy L, Kinney M, Schwartz FN (2015). Student perceptions of gamified audience response system interactions in large group lectures and via lecture capture technology. BMC Med Educ.

[10] Gupta A, Bhatti K, Walia R, Agnihotri P, Kaushal S (2015). Implementation of interactive teaching learning methods in large group in endocrine pharmacology. Indian J Pharm Pharmacol.

[11] Moellenberg KK, Aldridge M (2010). Sliding away from PowerPoint: the interactive lecture. Nurse Educ.

[12] The University of Arizona (2020). The Interactive Lecture. An Instructor’s Manual. https://fid.medicine.arizona.edu/sites/default/files/u4/interactivelecturemanual_omse.pdf.

[13] Madona G, Marine D (2017). Interactive teaching methods: challenges and perspectives. Int E-J Adv Educ.

[14] Kaur D, Singh J, S S, Mahajan A, Kaur G (2011). Role of interactive teaching in medical education. Inter J Basic Appl Med Sci.

[15] Begum J, Ali SI, Panda M (2020). Introduction of interactive teaching for undergraduate students in community medicine. Indian J Community Med.

[16] Verma A, Patyal A, Meena JK, Mathur M, Mathur N (2021). Interactive teaching in medical education: experiences and barriers. Adesh Univ J Med Sci Res.

